# Cross sectional, qualitative thematic analysis of patient perspectives of disease impact in juvenile idiopathic arthritis-associated uveitis

**DOI:** 10.1186/s12969-017-0189-6

**Published:** 2017-08-04

**Authors:** Ethan S. Sen, Michelle J. Morgan, Rachael MacLeod, Helen Strike, Ann Hinchcliffe, Andrew D. Dick, Brinda Muthusamy, Athimalaipet V. Ramanan

**Affiliations:** 1 Department of Paediatric Rheumatology, Bristol Royal Hospital for Children, Upper Maudlin Street, Bristol, BS2 8BJ UK; 2 School of Clinical Sciences, University of Bristol, Bristol, UK; 3 Sirona Care & Health, Community Children’s Health Partnership, Bristol, UK; 4 Department of Paediatrics, Bristol Royal Hospital for Children, Bristol, UK; 5 Retinal Treatment and Research Unit, Bristol Eye Hospital, Bristol, UK; 6 National Institute for Health Research Biomedical Research Centre at Moorfields Eye Hospital NHS Foundation Trust and UCL Institute of Ophthalmology, London, UK; 7 Department of Ophthalmology, Cambridge University Hospitals NHS Foundation Trust, Cambridge, UK

**Keywords:** Juvenile idiopathic arthritis, Uveitis, Quality of life, Patient-reported outcome measures

## Abstract

**Background:**

Chronic health conditions in children can have a significant impact on their quality of life. The aim of this study was to explore the subjective experience of children and young people being treated for chronic, non-infectious uveitis associated with a systemic disease such as juvenile idiopathic arthritis.

**Methods:**

A semi-structured interview was conducted with 10 children and young people aged between 6 and 18 years of age and their parents.

**Results:**

Preliminary thematic analysis indicated that both the treatment and complications of the disorder have a significant impact on the quality of life and emotional well-being of patients, not only in terms of the discomfort experienced but also in perceptions of social isolation, anxiety and sense of injustice.

**Conclusion:**

This study shows that themes including “impact on school”, “social factors” and “emotional reactions” are important domains influencing health-related quality of life (HRQoL) in children with chronic uveitis. Inclusion of questions relating to these domains should be considered in future uveitis-specific tools examining HRQoL in these patients.

## Background

The importance of measuring health-related quality of life (HRQoL) in children with chronic health conditions is widely acknowledged [1, 2]. Multiple generic, organ- and disease-specific patient-reported outcome measures (PROMs) of HRQoL in children exist, including tools that combine generic and specific measures [3–8]. HRQoL is known to result from a complex interaction of physical, social and cultural factors, with severity of illness or disability not necessarily being associated with worse HRQoL, as exemplified by the disability paradox [3, 9, 10].

Understandably, the HRQoL of children with visual impairment can be significantly reduced [3, 4, 6]. In recent years, a number of vision- and disease-specific HRQoL PROMs for children have been developed, beginning to populate a landscape that previously was largely void of such tools, although it is still felt that there is a lack of instruments to measure vision-related function and QoL in children overall [3, 4, 6–8, 11, 12]. Amongst these tools is the Effects of Youngsters’ Eyesight on Quality of Life Questionnaire (EYE-Q), which has been validated as a uveitis-specific measure, both for uveitis alone, and uveitis associated with chronic illness, such as juvenile idiopathic arthritis (JIA) [4]. The EYE-Q comprises 19 items, including 4 regarding QoL.

Chronic anterior uveitis (CAU), the persistent inflammation of the anterior segment of the eye, is the most frequent extra-articular manifestation of JIA. Often clinically silent, CAU can lead to visually-disabling complications including cataracts, glaucoma, band keratopathy and macular oedema, both as a result of chronic disease activity and treatment, which can be burdensome [13, 14]. Treatment includes topical and systemic glucocorticoids as well as systemic immunosuppressive therapies, including methotrexate, and newer biological therapies, with a move to introducing these sooner to minimise glucocorticoid use [14, 15].

This study aims to develop an understanding of the subjective experience of the impact of both uveitis treatment and its complications on the HRQoL of children, incorporating parent perceptions, with a view to ascertaining if any important additional topics are worth considering for inclusion in future PROMs of QoL in children with uveitis.

## Methods

This cross-sectional interview study was sponsored by the University Hospitals Bristol NHS Foundation Trust and approved by the South West Research Ethics Committee (Reference [11]/SW/0109, Protocol number CH/2010/3587, IRAS project ID 61606). This study included both child and parent perspectives, as there is often discordance between the two, with the parent proxy of cognitive and emotional reactions to various disorders differing from child reports [1, 16]. Both the children and their parents provided written informed consent/assent to participate in the study.

Ten subjects aged 6 to 18 years old and their parents were selected by purposive sampling from the records of the multi-disciplinary paediatric rheumatology and uveitis clinic. The sample was stratified by age, gender and uveitis severity in order to achieve a representative group for analysis. Two age bands were used for sampling: 6 to 12 years and 13 to 18 years with five participants in each age band. Five participants were male and five were female. The two categories of disease severity (moderate and severe uveitis) were equally represented in the sample. The severity of the uveitis was determined by the treating ophthalmologist using the Standardisation of Uveitis Nomenclature (SUN) anterior chamber (AC) cell grade, AC flare and vitreous haze together with ocular co-morbidities and the number of systemic therapies in preceding years [17, 18]. Nine children had JIA-associated uveitis and one child was diagnosed with Blau syndrome and uveitis. Children under 6 years old and those with significant learning difficulties were excluded due to restriction in cognitive and verbal skills [16].

A Consultant Clinical Psychologist conducted a 30 min, audio-taped, semi-structured interview covering six principal domains and a general section with each participant. The six domains included: treatment received, ocular complications, impact on school activities, impact on out-of-school activities, social impact and emotional reactions. The general section comprised further open-ended questions to elicit items that either the children or their parents felt were indicative of the impact of the disease on the child’s life but had not been previously covered. Whenever possible, children and parents were interviewed separately with the interview schedules covering the same domains but with the wording of questions adapted to the appropriate developmental level.

The audiotapes of the discussions were transcribed verbatim. Responses were analysed using inductive thematic analysis [19–21]. First the data were read carefully to identify meaningful units of text relevant to the research topic. Second, units of text dealing with the same issue were grouped together in analytic categories and given provisional labels. Finally, the data were reviewed to ensure that sets of data to support each label were identified.

## Results

A total of 10 children / young people were included in this study. Three patients, at their request, were interviewed together with their parents. In the remaining seven cases, the patients and parents were interviewed separately. The results, therefore, represent the outcomes from 17 different interviews.

Patient demographics and disease characteristics are shown in Table 1. As expected from the purposive sampling strategy, males and females, and ages above and below 12 years, were equally represented. The duration of uveitis ranged from 1.5 to over 8 years. There was a variety of disease severity: one patient had uncomplicated disease controlled with topical corticosteroid drops and subcutaneous methotrexate; other patients had ongoing active disease, previous treatments with four or five systemic therapies and surgery for cataracts or glaucoma.

**Table 1:** Patient demographics, immunosuppressive treatments and disease characteristics at the time of interview

Gender	Age (years)	Diagnosis	Duration of arthritis / uveitis (years)	Previous treatment	Treatment at time of interview	Active joints	Uveitis complications	Ophthalmic surgery	Eye	Visual acuity (LogMAR)	Anterior chamber cells (SUN grade)
M	6	Extended oligo JIA Left anterior uveitis	6 / 3.5	TS, MTX sc, ETA (stopped after onset of uveitis), ADA	MTX sc, ADA	nil	Posterior synechiae, cataract, band keratopathy	Orbital floor steroid injection	RE	0.000	0
									LE	0.150	0.5
F	7	RF-ve poly JIA Bilateral anterior uveitis	5 / 1.5	TS, MTX sc	MTX sc, ADA	nil	Glaucoma	Nil	RE	0.000	0
									LE	0.050	0
F	7	Persistent oligo JIA Bilateral anterior uveitis	2 / 2	TS, MTX sc	TS, MTX sc	nil	nil	nil	RE	−0.100	0
									LE	−0.100	0
F	10	Extended oligo JIA Bilateral anterior uveitis	5 / 5	TS, IVMP, MTX, MMF, INF, ADA	TS, MMF, ADA	nil	Posterior synechiae cataract, glaucoma	Cataract surgery BE, glaucoma surgery BE	RE	0.075	0
									LE	0.025	0.5
F	10	RF-ve poly JIA Bilateral anterior uveitis	9 / 5	TS, MTX sc, IVMP, INF, MMF, ADA	TS, MTX sc, ADA	Left ankle	Posterior synechiae, cataract, glaucoma, CMO, band keratopathy	Cataract surgery BE, Trabeculectomy BE	RE	0.125	0.5
									LE	0.175	0.5
M	13	RF-ve poly JIA Bilateral anterior uveitis	>8 / 6	IVMP, INF, MTX sc, ADA, MMF, ABA	MTX, MMF, ABA	nil	Posterior synechiae, cataract	Cataract surgery RE	RE	0.050	2
									LE	−0.100	3
F	14	Extended oligo JIA Bilateral anterior uveitis	>8 / >8	TS, MTX sc, INF	TS, MTX sc, ADA	Left hip, right knee	Cataract, glaucoma	Cataract surgery BE, glaucoma surgery BE	RE	−0.100	0.5
									LE	HM	0.5
M	16	Blau syndrome Bilateral panuveitis	15 / 8	TS, MTX sc, ADA	TS, MTX sc, MMF, IVMP, INF	nil	Posterior synechiae, cataract, chorioretinal scars, vitritis	nil	RE	0.850	1
									LE	0.100	1
M	16	Psoriatic JIA Bilateral anterior uveitis	>8 / >8	MTX sc, INF	MTX sc, ADA	nil	nil	nil	RE	0.000	0
									LE	−0.075	0
M	17	Extended oligo JIA Bilateral panuveitis	9 / 8	TS, MTX sc	TS, MTX sc	nil	Cataract, glaucoma	ST steroid injection RE	RE	0.125	0.5
									LE	−0.200	2

*Legend*: *ABA* Abatacept, *ADA* Adalimumab, *BE* Both eyes, *CMO*, Cystoid macular oedema, *ETA* Etanercept, *HM* Hand motion, *INF* Infliximab, *IVMP* Intravenous methylprednisolone, *JIA* Juvenile idiopathic arthritis, *LE* Left eye, *MMF* Mycophenolate mofetil, *MTX* Methotrexate, *oligo* Oligoarticular, *poly* Polyarticular, *RE* Right eye, *RF* Rheumatoid factor, *sc* Subcutaneously, *ST* Sub-Tenon’s capsule, *SUN* Standardisation of Uveitis Nomenclature, *TS* Topical steroids

The study has identified multiple important themes across the six principal domains of “impact of treatment”, “complications”, “impact on school”, “impact outside of school”, “social factors” and “emotional reactions” (Table 2).

**Table 2:** Themes identified across the six domains in the semi-structured interview

THEME	Frequency (*n* = 17 interviews)
DOMAIN 1: Impact of treatment	
Negative emotional reactions to treatment	10
Missing school for treatment	7
Anxiety about improvement	5
Avoidance	5
Side Effects	5
Anticipatory nausea	4
Painful Injections	4
Difficulty swallowing tablets	3
DOMAIN 2: Complications	
Cataract removal	6
Poor vision/blurring	6
Glaucoma surgery	3
DOMAIN 3: Impact on School	
Difficulties reading information	9
Upset at missing lessons	8
Teachers forgetting	7
Anxiety about catching up with missed work	5
Difficulties seeing the ball in PE	4
Upset at missing friends/activities at school	4
DOMAIN 4: Impact outside school	
Needing adaptations in order to participate	5
Not able to see bus numbers readily	4
DOMAIN 5: Social Factors	
Being excluded	8
Not chosen as team member	6
Bullying	4
Self-exclusion	2
DOMAIN 6: Emotional Reactions	
Distress/sadness about illness	11
Fear	9
Anger	8
Worry about deterioration	7
Worry about going blind	7
Worry about the future/careers	6
Why me?	2

In the “impact of treatment” domain, the direct effects of therapeutic interventions such as painful injections, nausea and difficulties swallowing tablets were highlighted. These, in turn, led to both anticipatory nausea in some patients and avoidance behaviour in others, with negative emotional reactions to treatment being a frequently occurring theme:

“On some occasions it would take up to two hours to administer the injection because he would cry and scream and throw a general wobbly…he ended up developing a phobia of all things yellow because the sharps bins were yellow and methotrexate is yellow”.

The indirect effects of receiving treatment, such as missing school for appointments and treatment were important to both children and young people. Additionally, anxiety about perceived lack of symptom improvement despite treatment was identified as a significant concern by one patient:

“I can get agitated as I don’t really see it getting any better”.

This lack of perceived improvement also exacerbated avoidance of treatments and caused difficulties between parents and children regarding adherence to treatment regimes. Patterns of non-adherence followed by “nagging” on the part of the parent were described. Unfortunately, adjustment to medication does not necessarily follow and some families indicated that the negative emotional reaction actually increased over time:

“She has got more upset about having her injection than she used to be”.

There were a number of themes identified in the “impact on school” domain. Upset at missing lessons and subsequent anxiety was a strong theme:

“I usually miss out on the understanding of some of the lessons because I wasn’t there for it – it makes you feel worried because I don’t know what I am doing”.

The pressure to catch up with missed schoolwork is an additional stress for these children, and parents are aware that progress can be patchy, often in relation to the child’s interests and motivation. There were also practical issues for those experiencing visual problems such as not being able to read the board in class, especially if placed at the rear of the classroom, or not being able to participate fully in Physical Education (PE):

“I’m always the last one to get ready for PE and everyone else is waiting on the carpet for me. I feel embarrassed and lonely”.

Even when adaptations are made by the school, they are not always successful, with young people commenting on laptop computers not being available at the beginning of each school year or examinations being printed on large sheets of paper that would not fit onto the desk. Furthermore, young people felt that teachers did not always remember the visual difficulties they had and they were reluctant to inform or remind teaching staff as they did not wish to appear different from their peers:

“I don’t really tell the teachers about my medical stuff ........I don’t want to sit in another place to other people because that can be embarrassing”.

There were fewer themes identified in the “impact outside of school” domain. The most commonly reported adaptations were sitting close to the television and holding hand held consoles or books close to the face. The zoom function on many IT tablets was reported as being very useful. Children and young people reported not being able to see the numbers on buses until they were quite close. Occasionally activities were dropped due to problems with eyesight which saddened young people.

In the “social factors” domain, the impact of both the treatment and complications of uveitis ranged from not feeling included or being the last person selected for a team or activity, to being actively bullied:

“I was bullied every day in Year 9 [age 13-14 years]...They found out I had had eye surgery and they called me ‘eye surgery gone wrong’. They said I was diseased or contagious and wouldn’t go near me”.

In addition to peer rejection, self-exclusion from activities was also reported. In older teenagers, restrictions imposed by treatment also led to feelings of being left out, especially when medication contraindicated alcohol consumption.

A range of almost universally negative emotional reactions both to the treatment and complications of uveitis were described. Reactions included anger, fear, embarrassment, isolation and low mood.

“…when I used to go to [hospital] for my cataracts and see the people in there and I was the youngest by at least half a century ...it gets a bit depressing”.

Much of the anxiety and worry surrounded possible visual deterioration and the unpredictability of the disease. Fears about the future were apparent, especially in relation to possible careers. Those who had identified potential careers for themselves were particularly concerned about the impact that eyesight problems would have. Some children and young people were explicit about their fear of going blind:

“I am scared I am going to go blind”.

Many of the children and young people involved in the study expressed distress, anger and resentment of the condition. There were reports of being frustrated and angry about the restrictions and limitations imposed by the condition, feelings often associated with questions about why this had happened to them. In more extreme cases, children and young people reported feeling that life was not worth living.

“I used to feel, well I still feel like ‘why me?’ and all that kind of stuff, because it can feel unfair”

## Discussion

Our study has identified multiple important themes across all 6 domains. As expected, within Domain 1, “impact of treatment”, the greatest number of themes for any single domain were identified. The themes most strongly represented were negative emotional reactions to treatment, missing school for treatment, side effects and treatment avoidance. Within Domain 2, “complications of uveitis and treatment”, the themes most frequently occurring were concerns regarding cataract removal and poor vision or visual blurring.

Domain 3, “impact on school”, also highlighted several important themes, primarily difficulties reading information, upset at missing lessons, teachers forgetting about their condition, anxiety about catching up with missed work and upset at missing friends. It was also apparent that it is more difficult for children to spend free time catching up with subjects that they dislike, leading to additional resentment about missed school. Furthermore, for many children and young people, school is the main opportunity to socialise with their peers, and interruption of peer interaction was raised as an important concern, as was re-integrating into the peer group following periods of absence. The social importance of school was demonstrated in several strong themes coming from Domain 5, “social factors”, including bullying, being excluded and not being chosen as a team member. Such bullying can lead to school avoidance, potentially further impairing the academic achievements of a young person already regularly absent from school due to hospital appointments and illness.

The “impact outside of school”, Domain 4, developed fewer themes, with the requirement of adaptations in order to participate in activities and the inability to see bus numbers with ease being identified.

In view of the distress and difficulty apparent from the themes discussed above, it is unsurprising perhaps that Domain 6 “emotional reactions” produced some very strong themes, primarily distress and sadness about the illness, fear, anger and worry about deterioration, going blind and the future. This implies that although specific practical and social difficulties are problematic, the combination of these issues, compounded by the frightening nature of a vision-threatening condition, can result in a heavy emotional burden.

The only current validated uveitis-specific QoL measure in children, the EYE-Q, comprises 19 items measuring near, far, colour and night vision, photosensitivity, and functionality**.** There are four QoL items enquiring about feelings regarding the use of medications, missing school for appointments, and “laboratory draws”. One question enquires about the presence of common uveitis symptoms, and there is an item that enquires about visual aids [4]. An early version was felt to be more a measure of visual function than QoL, demonstrated by EYE-Q scores being weakly associated with HRQoL measured using the generic PedsQL tool, however the most recent version, which included the 4 QoL questions, shows moderate correlation with PedsQL [4, 11, 22]. The topics include two important issues identified in Domain 1, “impact of treatment”, one from Domain 2, “complications” and one from Domain 4 “impact outside school”. However, no themes from Domains 3, 5 or 6, “impact on school”, “social factors” and “emotional reactions” are covered, topics we found to be of significant concern to children in our study.

Other tools, generic, vision and disease-specific, measuring QoL in children have good coverage of these important themes (Table 3).

**Table 3:** Coverage of QoL domains by tool

	Quality of Life tool					
Domain	EYE-Q [4]	Peds-QL [5]	IVI-C [8]	VQoL-CYP [3]	ITXQ [23]	QUICK [7]
1: Impact of treatment	X					
2: Complications	X					
3: Impact on school		X	X	X		
4: Impact outside school	X		X	X		
5: Social factors		X	X	X	X	X
6: Emotional reactions		X	X		X	

*Legend*: *EYE-Q* Effects of Youngsters’ Eyesight on Quality of Life Questionnaire, *ITXQ* Intermittent exotropia questionnaire, *IVI-C* Impact of Vision Impairment for Children, *PedsQL* Pediatric Quality of Life Inventory, *QUICK* Quality of Life in Children with Vernal Keratoconjunctivitis, *VQoL_CYP* Vision-related Quality of Life Instrument for Children and Young People

The PedsQL, a validated measure of general HRQoL in children aged 2–18 years, covers 4 core scales: physical functioning, emotional functioning, social functioning and school functioning, with 5 questions in each [5]. It thoroughly covers three domains we also found to be important: “impact on school”, “social factors” and “emotional reactions”. It is the standard against which other QoL tools are correlated [3, 4, 11].

The Impact of Vision Impairment on Children (IVI_C) is a 24 question, psychometrically valid vision-specific QoL measure, for use in vision-impaired children aged 8 to 18 years who have no additional disabilities. It includes themes identified in Domains 3, 4, 5 and 6, with a heavy emphasis on the impact on school and social functioning. It has been shown to have construct, face and content validity, as well as internal consistency and good test re-test reliability [8, 11]. A second vision-specific instrument, the VQoL_CYP, is an age-appropriate, vision related quality of life instrument for self-reporting by children with visual impairment, consisting of a 35 item scale [3]. It covers Domain 3 and has extensive coverage of Domains 4 and 5, with a focus on “social factors” and “emotional reactions” in particular. A strong correlation between VQoL_CYP and PedsQL Total Summary Score, particularly its psychosocial health scale has been shown. The correlation of VQoL_CYP with the PedsQL psychosocial summary score was found to be greater than the moderate correlation with PedsQL physical health summary score, providing evidence for strong psychosocial component of the VQoL_CYP [3].

A disease-specific tool, the Intermittent Exotropia Questionnaire (ITXQ), is a 3-part, patient-derived HRQoL questionnaire for children with intermittent exotropia (IXT) and their parents, comprising child, proxy and parent questionnaires with 12 questions in each part [23]. It especially examines “emotional concerns”, Domain 6, and some “social factors”, Domain 5. Although the ITXQ was not directly compared with another measure of HRQoL, during validity studies, children with ITX were shown to have worse HRQoL than controls. This finding is reinforced by previous studies showing children with ITX had worse HRQoL compared to controls, when measured by PedsQL [23]. A second disease-specific tool, the Quality of Life in Children with Vernal Keratoconjunctivitis (QUICK) questionnaire is for children aged 5 to 12 years with chronic keratoconjunctivitis [7]. It consists of 16 items and primarily enquires about symptoms and the social impact of disease, Domain 5. It showed internal validity and consistency, with significant correlations to the KINDL, a generic instrument for assessing HRQoL in children and adolescents aged 3 years and older and an alternative to PedsQL [24].

This study has included patients of a variety of ages and with a range of uveitis disease severity. Patients were recruited through a joint Paediatric Rheumatology / Ophthalmology clinic at a tertiary referral hospital and those included had moderate or severe disease. The results may not, therefore, be representative of patients with mild, uncomplicated disease controlled with a single agent. One included patient had Blau syndrome with panuveitis, predominantly anterior uveitis. One of the patients with JIA-associated uveitis had panuveitis and another also had macular involvement (Table 1). It is unlikely that the impact of uveitis and its treatment in the patient with Blau syndrome is very dissimilar to those with JIA.

The study has identified themes within three domains (“impact on school”, “social factors” and “emotional reactions”) which are affected specifically by uveitis disease and treatment but are not covered by the EYE-Q. Questions which address these domains are included in other generic and ophthalmology disease-specific HRQoL tools for children (Table 3). In the future, it will be important to develop uveitis-specific HRQoL tools covering all these domains, and to include patients with other similar diseases and healthy controls as part of the validation process.

Clinical trials of treatments for JIA-associated uveitis are ongoing, including APTITUDE, a phase II trial of tocilizumab in anti-tumour necrosis factor (TNF) refractory patients [25] and studies of other biologic drugs are likely in the coming years. HRQoL measures are increasingly important as part of health economic evaluation of novel treatments [26]. These form a critical part of the recommendations of organisations advising health services about clinical and cost effectiveness such as the National Institute for Health and Care Excellence (NICE) in the UK. Similarly, being able to demonstrate effectiveness of treatment of JIA-associated uveitis using not only SUN criteria but also PROMs and HRQoL will provide additional patient-centred outcomes [27].

## Conclusion

Multiple vision and disease-specific measures of HRQoL include questions on domains that this study has shown to be important factors influencing HRQoL in children with chronic anterior uveitis, such as “impact on school”, “social factors” and “emotional reactions”. This study suggests that the inclusion of these domains should be considered in future uveitis-specific tools examining HRQoL in children.

## Abbreviations

CAU: Chronic anterior uveitis
CVFQ: Children’s Visual Function Questionnaire
EYE-Q: Effects of Youngsters’ Eyesight on Quality of Life Questionnaire
HRQoL: Health related quality of life
ITX: Intermittent exotropia
ITXQ: Intermittent exotropia questionnaire
IVI-C: Impact of Vision Impairment for Children
JIA: Juvenile idiopathic arthritis
PedsQL: Pediatric Quality of Life Inventory
PROM: Patient-reported outcome measure
QoL: Quality of life
QUICK: Quality of Life in Children with Vernal Keratoconjunctivitis
SUN: Standardisation of uveitis nomenclature
VQoL_CYP: Vision-related Quality of Life Instrument for Children and Young People
